# Regulation of microglial activation in stroke in aged mice: a translational study

**DOI:** 10.18632/aging.204216

**Published:** 2022-08-12

**Authors:** Conelius Ngwa, Abdullah Al Mamun, Shaohua Qi, Romana Sharmeen, Yan Xu, Fudong Liu

**Affiliations:** 1 Department of Neurology, The University of Texas Health Science Center at Houston, McGovern Medical School, Houston, TX 77030, USA

**Keywords:** aging, inflammation, IRF, microglia, stroke

## Abstract

Numerous neurochemical changes occur with aging and stroke mainly affects the elderly. Our previous study has found interferon regulatory factor 5 (IRF5) and 4 (IRF4) regulate neuroinflammation in young stroke mice. However, whether the IRF5-IRF4 regulatory axis has the same effect in aged brains is not known. In this study, aged (18-20-month-old), microglial IRF5 or IRF4 conditional knockout (CKO) mice were subjected to a 60-min middle cerebral artery occlusion (MCAO). Stroke outcomes were quantified at 3d after MCAO. Flow cytometry and ELISA were performed to evaluate microglial activation and immune responses. We found aged microglia express higher levels of IRF5 and lower levels of IRF4 than young microglia after stroke. IRF5 CKO aged mice had improved stroke outcomes; whereas worse outcomes were seen in IRF4 CKO vs. their flox controls. IRF5 CKO aged microglia had significantly lower levels of IL-1β and CD68 than controls; whereas significantly higher levels of IL-1β and TNF-α were seen in IRF4 CKO vs. control microglia. Plasma levels of TNF-α and MIP-1α were decreased in IRF5 CKO vs. flox aged mice, and IL-1β/IL-6 levels were increased in IRF4 CKO vs. controls. The anti-inflammatory cytokines (IL-4/IL-10) levels were higher in IRF5 CKO, and lower in IRF4 CKO aged mice vs. their flox controls. IRF5 and IRF4 signaling drives microglial pro- and anti-inflammatory response respectively; microglial IRF5 is detrimental and IRF4 beneficial for aged mice in stroke. IRF5-IRF4 axis is a promising target for developing new, effective therapeutic strategies for the cerebral ischemia.

## INTRODUCTION

Numerous neurochemical and neurobiological changes occur with aging [1–3]. As a fundamental pathophysiological process in stroke, neuroinflammation also showed age-related differences [4–8]. Microglial activation plays a central role in initiating and perpetuating the post-stroke inflammation, and acts as a “double-edged” sword to confer both detrimental and beneficial effects [9]. Our previous studies using young animal stroke model have found a determinant role of IRF4-IRF5 regulatory axis in mediating microglial responses after stroke; however, whether aged microglia also undergo the same regulatory mechanism after ischemia has been elusive. Aged microglia are primed to activation even without any exogenous pathological stimulus [10]; once activated, they showed aberrant phagocytosis compared to the young microglia. Stroke is a disease that mainly affects the elderly. Therefore, it is of high translational value to study how aged microglia is regulated in stroke.

In this study, we used transgenic mouse models to specifically study the effect of conditional knockout (CKO) of IRF4 or IRF5 in microglia on post-stroke inflammation and outcomes. We have previously found IRF4 signaling is anti-inflammatory and IRF5 is pro-inflammatory in young ischemic microglia [11]. In the present study, we hypothesized IRF4 CKO worsens while IRF5 CKO improves stroke outcomes. By using the aged IRF4/IRF5 microglial CKO mouse models, the study aimed to selectively suppress microglial pro-inflammatory activation and promote its anti-inflammatory response, and will potentially help develop new, effective therapeutic strategies against stroke.

## RESULTS

### Microglial IRF5 and IRF4 were differently expressed in young and aged mice

**Figure 1:**
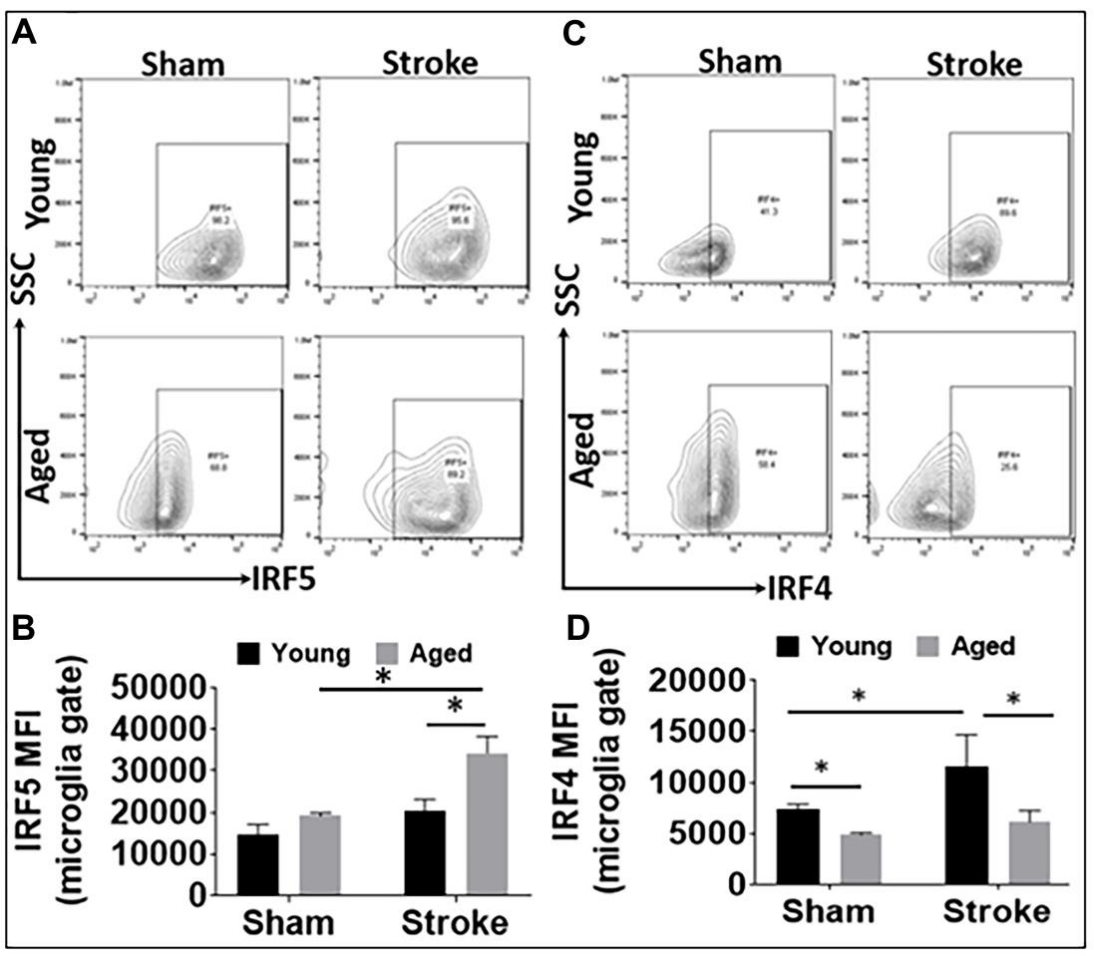
**IRF5 and IRF4 expression levels in microglia in young and aged C57BL/6 mice.** (**A**, **B**) Representative flow plots of microglial IRF5 (**A**) and the mean fluorescence intensity (MFI) (**B**). (**C**, **D**) Representative flow plots of microglial IRF4 (**C**) and the MFI) (**D**). n = 4-5 /group sham and 6-7/group stroke; *p<0.0500.

We have previously shown microglial phenotype differences in young and aged mice brains after stroke [4, 11]. In the present study, we further investigated microglial IRF5 and IRF4 expression in young vs. aged mice after stroke with flow cytometry. Microglia were gated as CD45^int^CD11b^+^ (see Supplementary Figure 1 for the gating strategy), and IRF5 or IRF4 mean fluorescence intensity (MFI) was quantified in these cells after 3d of MCAO (Figure 1). As expected, the 60 min MCAO induced a significant increase in the aged microglial IRF5 MFI, and the level was also higher in the aged vs. young ischemic microglia (Figure 1A, 1B). Contrary to this pattern, microglial IRF4 MFI in young mice was significantly higher than in aged mice in either the sham or the stroke groups; in addition, young microglial IRF4 significantly increased after stroke, an ischemic effect that was not seen in the aged cohort (Figure 1C, 1D).

### IRF deletion alters microglial inflammatory mediator expression in aged mice

**Figure 2:**
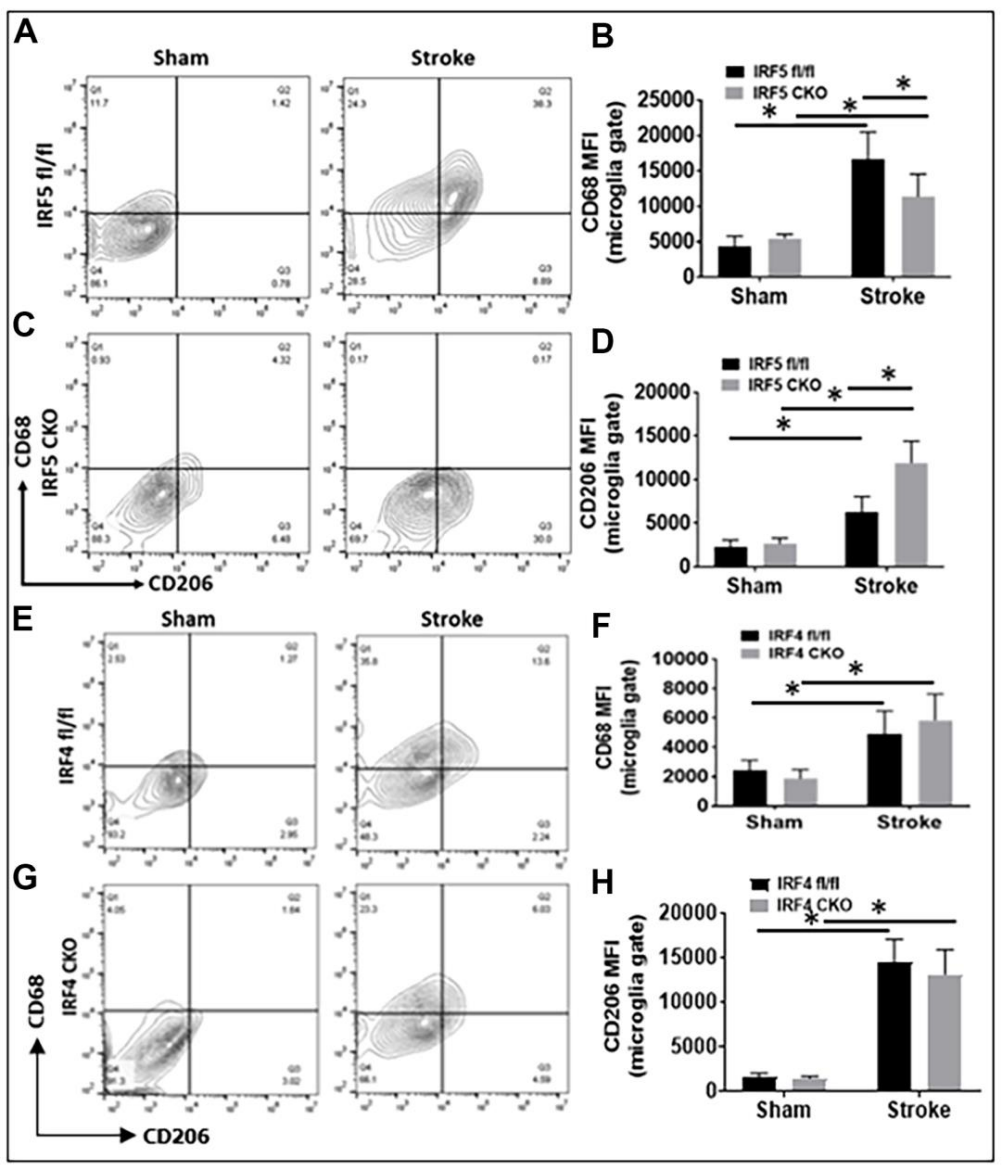
**Cell-membrane inflammatory marker levels in IRF5 or IRF4 CKO vs. flox microglia by flow cytometry performed on stroke and sham brains.** (**A**, **C**) Representative flow plots of IRF5 CKO, and (**E**, **G**) IRF4 CKO microglia gated by CD68 and CD206. (**B**, **D**, **F**, **H**) Quantification data of MFI for CD68 (**B** for IRF5, and F for IRF4) and CD206 (**D** for IRF5, and H for IRF4). *n* = 4 to 5 per sham and 6 to 7 per stroke group; *P < 0.0500.

Aging impacts on the immunological response to ischemic stroke [8]. To investigate whether IRF5 and IRF4 signaling induce similar inflammatory responses to stroke in aged microglia as that in the young [11, 12], we examined both surface and intracellular inflammatory markers in microglia from IRF5 or IRF4 CKO aged mice at 3d of MCAO. The CKO mice were generated by crossing IRF5 or IRF4 floxed mice with lysozyme M (LysM) Cre mice that have been widely used to target genes of interest in microglia [11, 13, 14]. CD68 and CD206 are well-established pro- and anti-inflammatory markers respectively expressed on microglial cell membrane [15–17]. Our results showed that MFI of CD68 was significantly downregulated in IRF5 CKO vs. flox mice after stroke; meanwhile CD206 was significantly upregulated by IRF5 CKO (Figure 2A–2D). CKO of IRF4 in aged microglia had no effect on CD68 or CD206 expression (Figure 2E–2H). Stroke caused significant increase in the two markers in all groups compared to shams.

**Figure 3:**
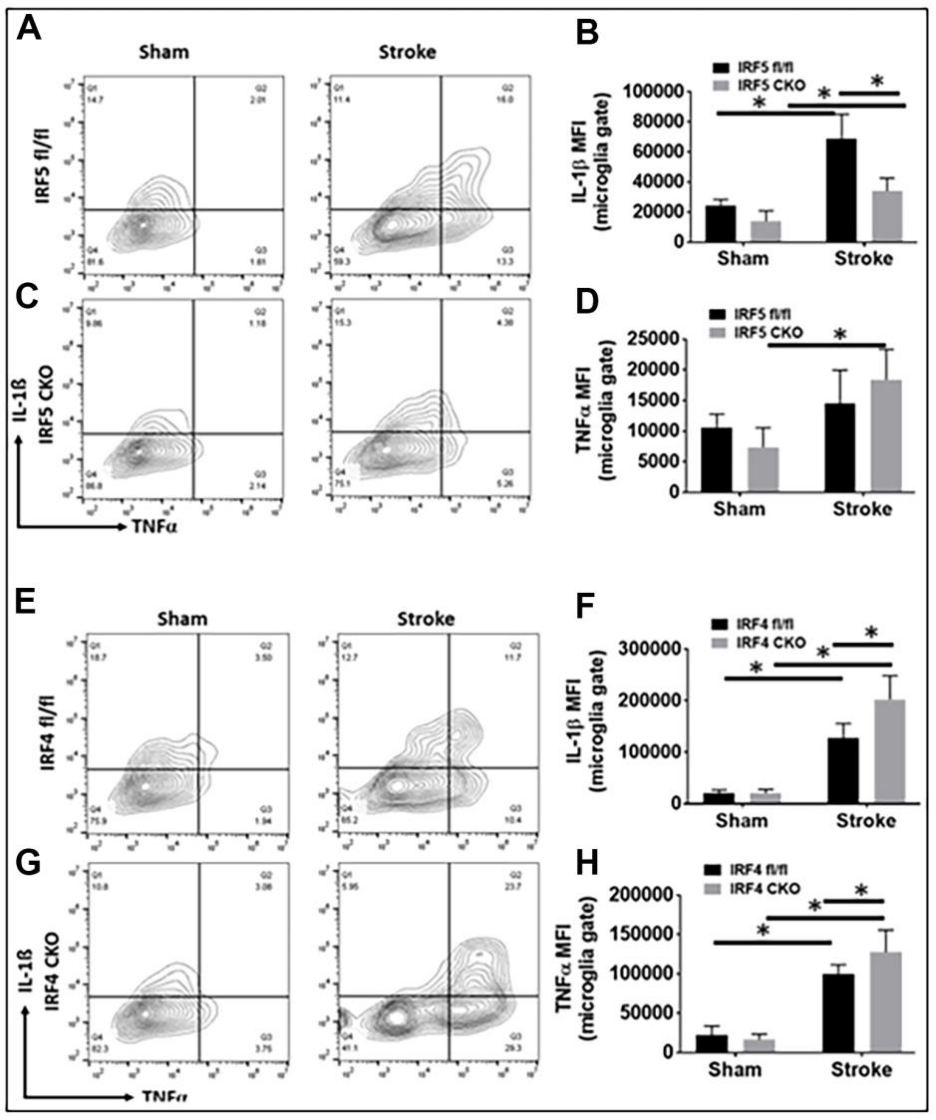
**Intracellular cytokine levels in IRF5 or IRF4 CKO vs. flox microglia by flow cytometry performed on stroke and sham brains.** Quantification data are presented as mean MFI. (**A**–**D**) Data of IRF5 CKO and (**E**–**H**) data of IRF4 CKO microglia, respectively. (**A**, **C**) are representative intracellular staining plots for IL-1β/TNF-α in both IRF5 CKO and flox microglia; (**E**, **G**) are plots for IL-1β/TNF-α in both IRF4 CKO and flox microglia. MFI of these cytokines were quantified in (**B**, **D**, **F**, **H**). *n* = 4 to 5 per sham and 6 to 7 per stroke group; *P < 0.0500.

For intracellular inflammatory markers we investigated IL-1β/TNF-α (pro-inflammatory) and IL-4/IL-10 (anti-inflammatory) [11]. While IRF5 CKO led to a decrease only in IL-1β expression after stroke, IRF4 CKO caused up-regulation of both IL-1β and TNF-α in ischemic microglia (Figure 3A–3H). Neither IRF5 nor IRF4 CKO had any effect on microglial IL-4 or IL-10 expression (Supplementary Figure 2A–2H). There were stroke effects on almost all these markers.

### Microglia mediated phagocytosis

**Figure 4:**
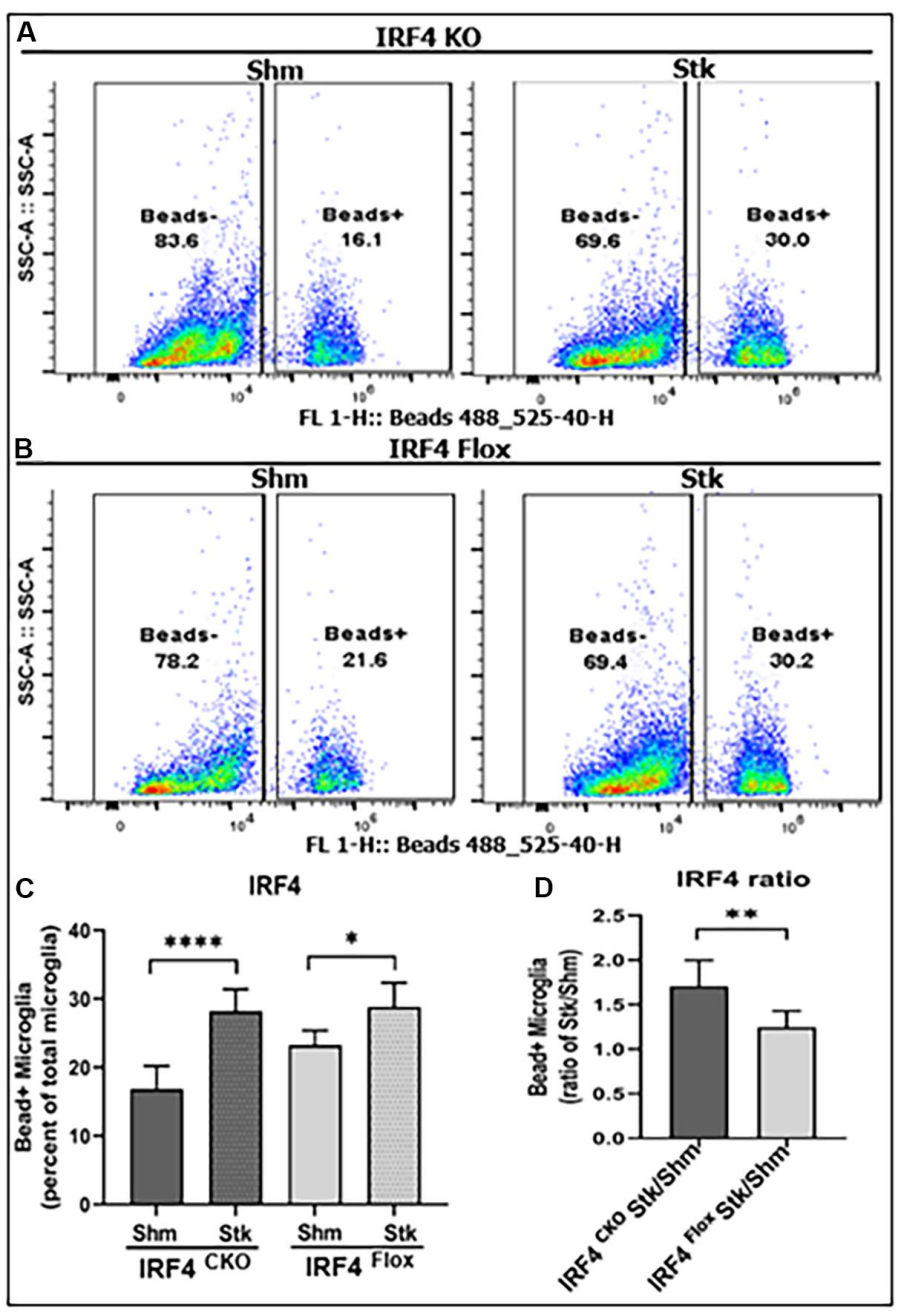
**Microglial phagocytosis by flow cytometry performed on IRF4 CKO stroke and sham mice.** Quantification data are presented as mean percentage of bead^+^ microglia. (**A**, **B**) Fluorescence intensity plots for IRF4 KO and flox microglia exposed to FITC fluorescent bioparticles. (**C**) Percentage fluorescence of phagocytosis in IRF4 CKO vs. flox sham and stroke microglia; and (**D**) comparative quantified data for the ratio of IRF4 CKO stk/shm vs. IRF4 flox stk/shm in (**C**). *n* = 4 to 5 per sham and 6 to 7 per stroke group; *P < 0.0500, **P < 0.0010, ***P < 0.0001.

Phagocytosis is a highly specialized process for the uptake and removal of opsonized and non-opsonized targets including pathogens, apoptotic cells, and cellular debris in all phagocytes including microglia [18, 19]. We already knew IRF5 and IRF4 signaling impact on microglial expression of cell membrane (Figure 2) and intracellular inflammatory markers (Figure 3). Next, we sought to investigate whether IRF5 or IRF4 also affect another important microglial function, i.e. phagocytosis, which was measured by the cell’s ability to phagocytose FITC bioparticles with flow cytometry in these aged mice. Figure 4A, 4B show the flow gating strategy for microglia that phagocytosed FITC bioparticles (Beads^+^) [5, 20]. After stroke, the ischemic microglia had increased phagocytosis in either IRF4 CKO or flox group (Figure 4C); however, the increase was significantly more robust in IRF4 CKO vs. flox mice as indicated by the ratios of stroke over sham group (Figure 4D). IRF5 signaling does not have any effect on the microglial phagocytosis (Supplementary Figure 3).

### Inflammatory mediator levels in plasma

**Figure 5:**
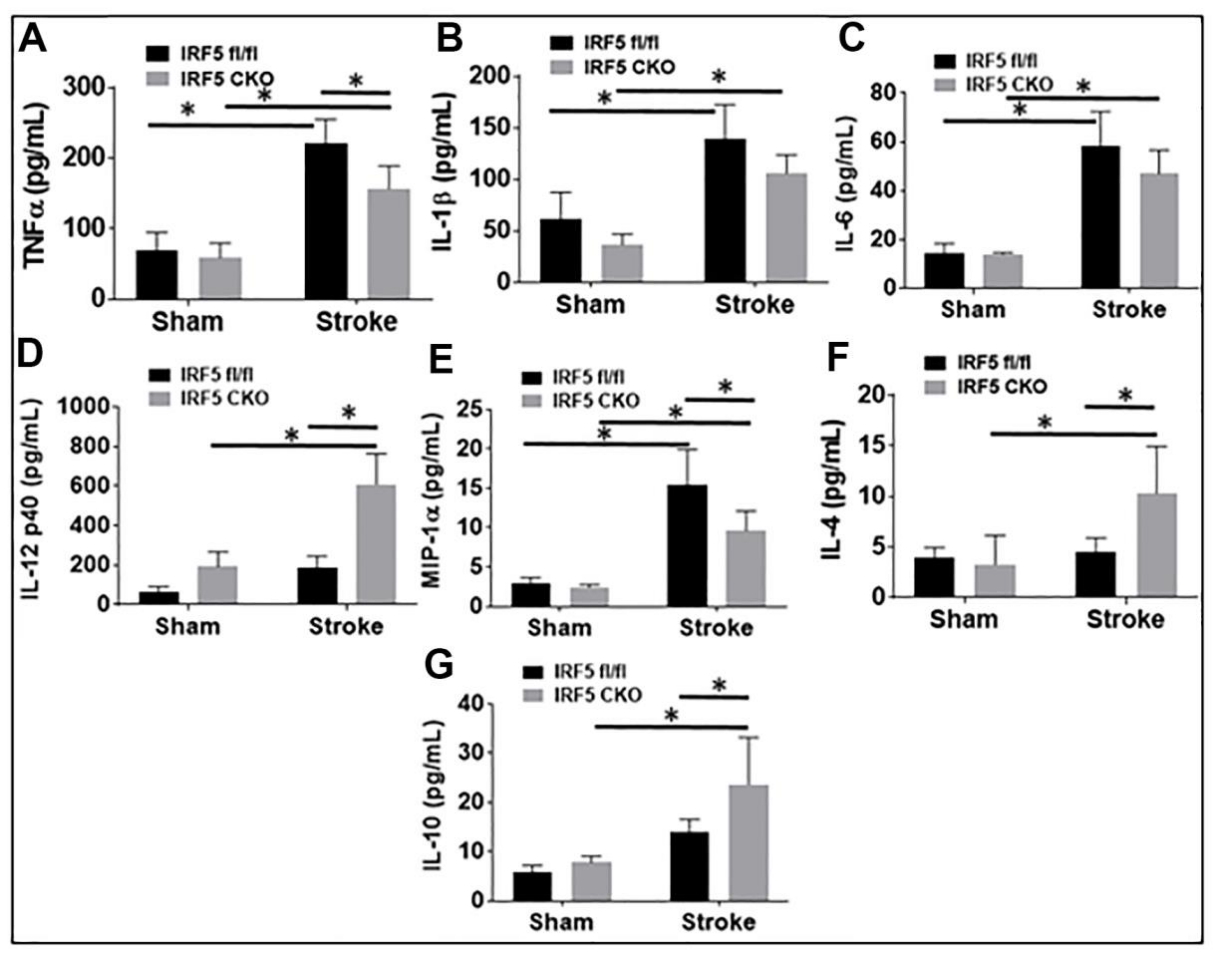
**IRF5 blood plasma levels of inflammatory mediators at 3d after MCAO.** Proinflammatory mediators (TNF-α, IL-1β, IL-6, IL-12p40, and MIP-α; **A**–**E**) and anti-inflammatory mediators (IL-4 and IL-10; **F**, **G**) in IRF5 CKO mice. Each sample was probed in duplicates. *n*=6 per stroke and 4 per sham group; *P < 0.0500.

**Figure 6:**
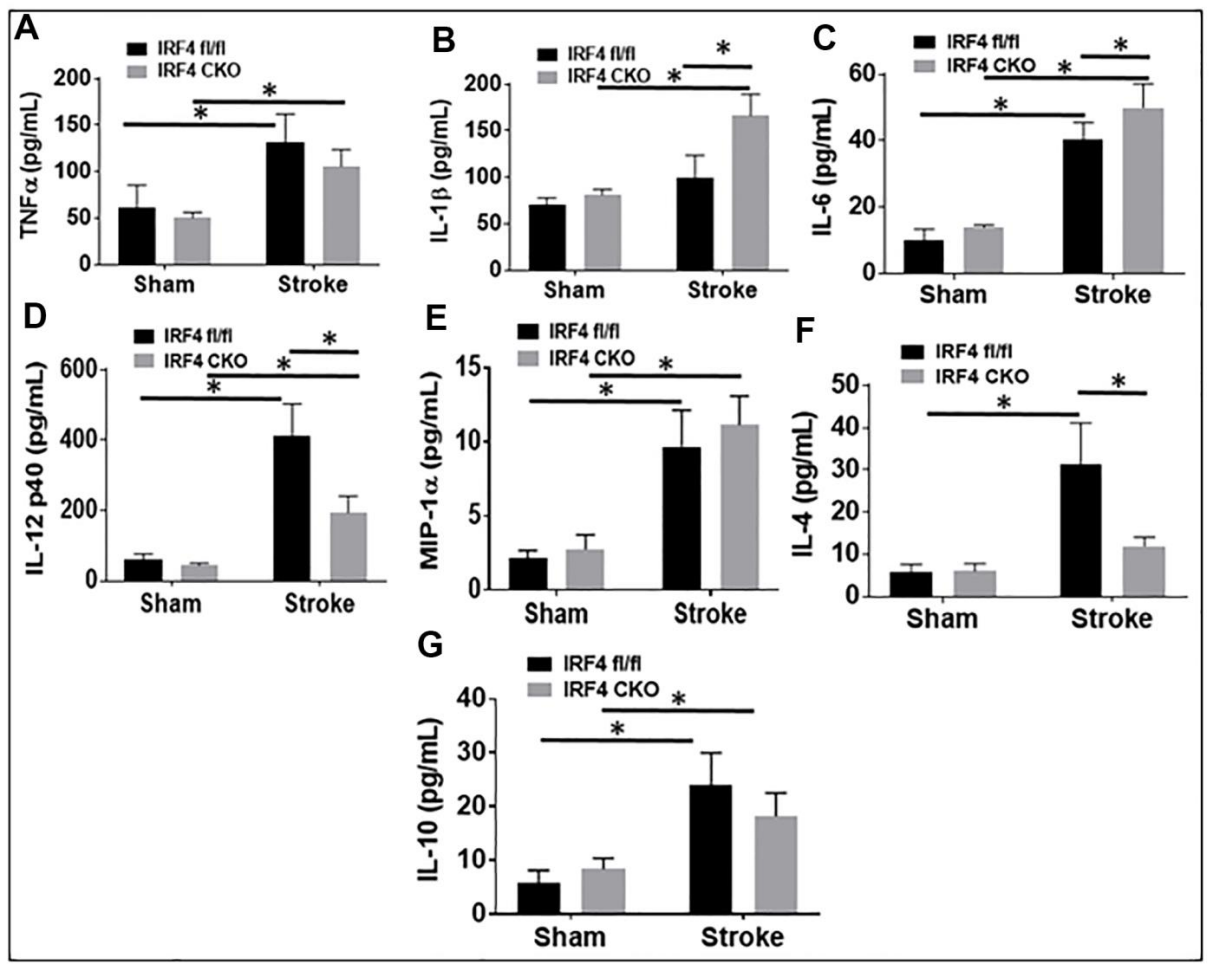
**IRF4 blood plasma levels of inflammatory mediators at 3d after MCAO.** Proinflammatory mediators (TNF-α, IL-1β, IL-6, IL-12p40, and MIP-α; **A**–**E**) and anti-inflammatory mediators (IL-4 and IL-10; **F**, **G**) in IRF4 CKO mice. Each sample was probed in duplicates. *n*=6 per stroke and 4 per sham group; *P < 0.0500.

Blood plasma cytokine levels are reflective of systemic inflammatory responses to stroke [21–23], and circulating cytokines direct the migration and infiltration of peripheral immune cells into the ischemic brain [24–27]. Since microglial activation triggers and perpetuates immune responses to stroke [28–31] and IRF5/IRF4 signaling regulate microglial activation, we next wanted to know if microglial IRF5 or IRF4 signaling impacts on plasma cytokine levels. The pro-inflammatory (TNF-α, IL-1β, IL-6, IL-12 p40, and MIP-1α), and the anti-inflammatory (IL-4 and IL-10) mediators were examined by ELISA in IRF5 or IRF4 CKO aged mice plasma, 3 days after stroke. As expected, stroke effects were evident as levels of almost all the inflammatory mediators significantly increased after stroke in either IRF flox or CKO groups (Figures 5, 6). However, when we compared the levels of pro-inflammatory mediators between flox and CKO mice after stroke, we found the differences within groups exhibited a heterogeneous manner (Figures 5A–5E, 6A–6E). TNF-α/MIP-1α levels significantly decreased, but IL-12p40 significantly increased in IRF5 CKO vs. flox mice after stroke (Figure 5A, 5E, 5D). IL-1β/IL-6 increased but IL-12p40 decreased in IRF4 CKO vs. flox mice after stroke (Figure 6B, 6C, 6D). For anti-inflammatory cytokines, a relatively homogenous pattern was seen; IRF5 CKO induced a significant increase in both IL-4 and IL-10 levels (Figure 5F, 5G), and IRF4 CKO caused significant reduction in IL-4 (Figure 6F) and a decrease trend in IL-10 (Figure 6G) level, in stroke groups.

### IRF5 is detrimental; IRF4 is protective in aged mice

**Figure 7:**
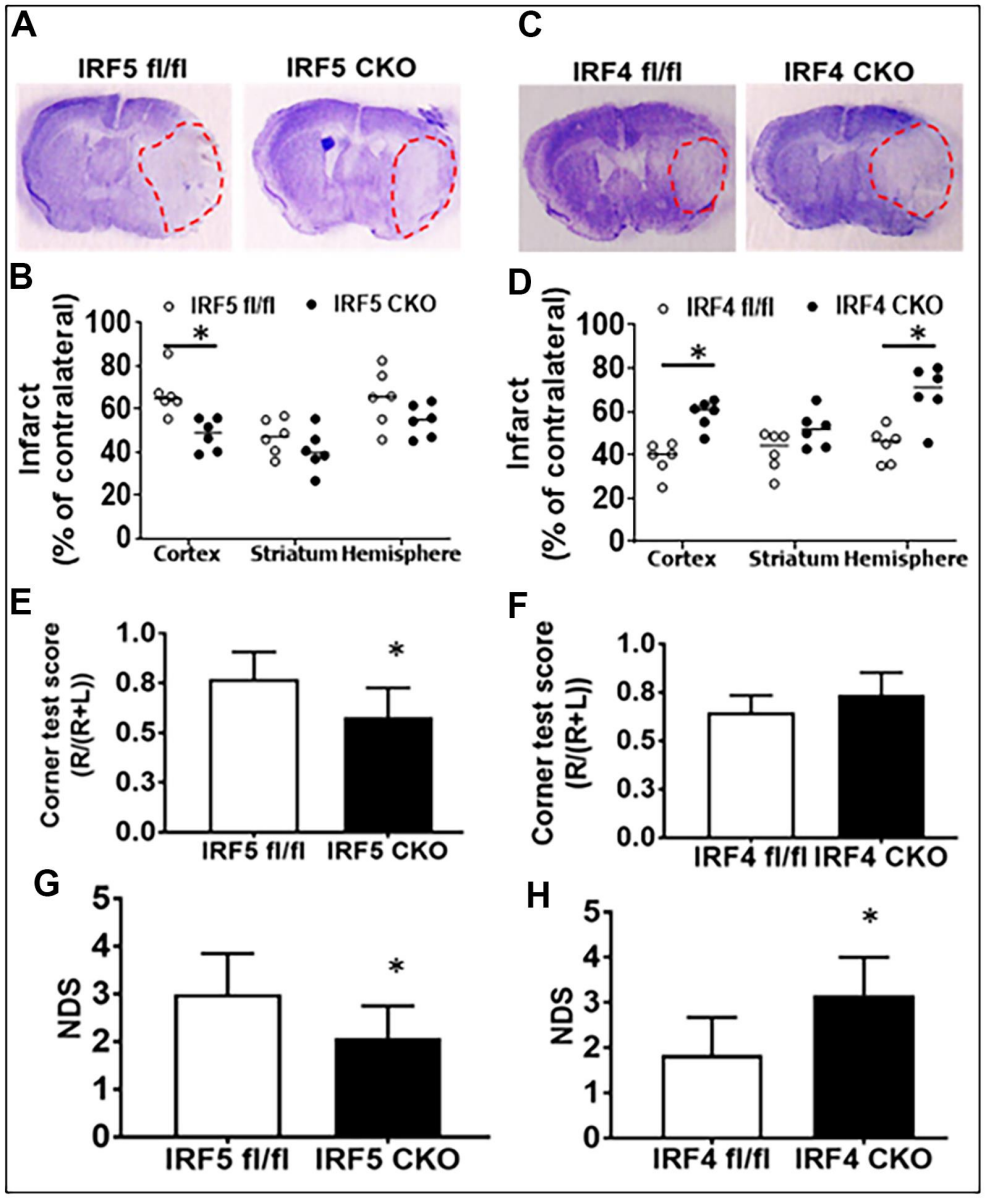
**Stroke outcomes in aged IRF5/4 CKO and flox mice at 3d after MCAO.** (**A**, **C**) Representative IRF5 and IRF4 CKO vs. flox brain slices stained with cresyl violet. (**B**, **D**) Quantification of infarct volumes. (**E**, **F**) Corner test scores calculated by R/(R+L) x100, where R and L are right and left turn number respectively. (**G**, **H**) Neurological deficits scores. n =6/group; *P < 0.0500.

Lastly, we evaluated stroke outcomes in these IRF5 or IRF4 CKO aged mice. Three days after MCAO, the infarct volumes and a battery of behavior tests were examined. We found significantly smaller infarcts in brain cortical regions of aged IRF5 CKO vs. flox (Figure 7A, 7B) mice. In contrast, IRF4 CKO led to significantly larger infarct in the cortex and the whole ipsilateral hemisphere (Figure 7C, 7D). Positive results were seen in two behavior tests: corner test and NDS. IRF5 CKO mice had better outcomes in both tests (Figure 7E, 7G), and IRF4 CKO led to significantly higher NDS (Figure 7H) after MCAO. These data suggest that IRF5 is detrimental and IRF4 protective in aged stroke.

## DISCUSSION

Aging causes neurochemical and physiological changes in brains. We have previously reported the roles of IRF5-IRF4 signaling in the activation of neonatal and young adult microglia after ischemia [11, 12, 32]. The present study focused on aged microglia, and examined the inflammatory responses and stroke outcomes in IRF5/4 CKO vs. flox aged mice. We found aged microglia express more IRF5 and less IRF4 compared to young microglia after stroke. Stroke effects on these IRFs’ expression were also age-dependent, as in aged microglia only IRF5 expression increased after stroke in contrast to IRF4 that only increased in young ischemic microglia. Nevertheless, the age-dependent expression of the two IRFs was not translated into an age-dependent role of the IRF5-IRF4 axis, as IRF5 CKO induced a beneficial and IRF4 CKO elicited a detrimental effect on stroke outcomes, similar to what we have seen in young and neonatal mice stroke models [11, 33]. Unlike the typical oscillating pattern of microglial activation regulated by IRF5-IRF4 regulatory axis seen in young mice [11], IRF5 modulated the expression of aged microglial cell membrane markers (CD68, CD206), a regulatory role that was not seen in IRF4 CKO mice. In addition, IRF4 signaling, but not IRF5, impacts on microglial phagocytosis significantly.

Ischemia induces inflammatory responses that are characterized by a series of events (microglial activation, cytokine release, etc.) that lead to infiltration of monocytes, neutrophils, and lymphocytes into the ischemic brain and exacerbate tissue damage [34]. Microglia and monocytes are the major source of inflammatory cytokines in neuroinflammation [35–37], and these cells are tightly controlled by IRFs to exhibit either pro- or anti-inflammatory phenotype in the ischemic brain [38, 39]. Of the nine different subtypes of IRFs known based on their binding motifs [40, 41], IRF5 and IRF4 have been reported to regulate macrophage activation [11, 32, 35, 42, 43]. Our previous studies have found IRF5 and IRF4 signaling also regulate microglial activation in neonatal and young adult mice after stroke, with IRF5 being pro-inflammatory and IRF4 anti-inflammatory [11, 33, 44]. These two IRFs form an IRF5-IRF4 regulatory axis to synergistically mediate microglial polarization after ischemic insults in an oscillating manner [11]. The present study used a more clinically relevant stroke model, i.e. aged mice MCAO, and has found partially similar results but with distinct age-related differences in IRF5-IRF4 signaling.

Our flow data showed IRF5 was significantly expressed in aged vs. young microglia after stroke, as opposed to IRF4 that demonstrated an opposite expression pattern in microglia of the two ages. Microglia from aged brains are primed to be activated [5, 10] due to the impairments in several key regulatory systems with age that make it difficult to resolve microglial activation. This might explain why the expression of cell membrane markers on aged microglia were regulated only by IRF5 but not by IRF4 (Figure 2); while in young microglia both IRF5 and IRF4 impact on the expression [11]. Among the intracellular cytokine markers in aged microglia, only the pro-inflammatory marker expression (Figure 3) was sensitive to IRF5-IRF4 regulatory axis which had no effect on the anti-inflammatory cytokines (Supplementary Figure 2). This is also different from that of young microglia whose pro- and anti-inflammatory intracellular markers were both regulated by the IRF5/IRF4 signaling [11]. Nevertheless, when we examined the plasma levels of cytokines, we found either pro- or anti-inflammatory cytokines were regulated by the axis in an oscillating manner. The inconsistency of the cytokine marker expression pattern in microglia vs. plasma by IRF5/IRF4 regulation might be due to the reason that our CKO mice model was made by Lysozyme Cre. Lysozyme is also expressed by monocytes (macrophages), and the IRF5/IRF4 regulatory mechanisms might be different in aged macrophages vs. microglia; therefore, the plasma cytokine levels reflect the combined effects of knockout (KO) of IRF5/IRF4 in aged microglia and macrophages (a caveat of the study). Ongoing experiments in the laboratory are using bone marrow chimera model [8] and inducible CKO animals based on CX3CR1-CreER mice [45] to specifically examine monocytic vs. microglial IRFs. Of note, in Figure 5 the so-called pro-inflammatory marker IL-12p40 exhibited a pattern that is the same as that in anti-inflammatory markers IL-4 or IL-10. However, it is very interesting that IL-12 has been found to have either pro- or anti-inflammatory activity depending on the blood pressure (BP) [46]. It was suggested that in hypertensive state, IL-12 is anti-inflammatory but becomes pro-inflammatory in normotensive state [47]. We did not measure the BP in our aged CKO mice which is also a caveat of the present study; however, these aged mice might have a different BP homeostasis than young mice and therefore the aged IL-12p40 was pre-set towards an anti-inflammatory response. Another caveat of the study is that we examined IRF5-IRF4 axis in aged microglia only at 3 days post-stroke, because the CKO of IRFs in microglia induced a very high mortality in the aged mice (>50%) when we performed experiments at chronic time points longer than 3 days. However, previous studies [12, 48–53] have shown post-stroke inflammation peaks at 3d of stroke; therefore, 3d post-stroke may represent the optimal time point to examine the effects of CKO of IRFs from microglia on post-stroke inflammation. Nevertheless, to study the effects of IRF5-IRF4 signaling on chronic stroke outcomes with appropriate animal models (e.g. young IRF5/IRF4 CKO mice) is warranted in the future.

Phagocytosis is an important characteristic of microglia and has been reported to exhibit different profiles in neonatal, young adult and aged microglia [5, 8, 32]. The phagocytic activity has been widely studied using a variety of fluorescence materials including fluorescent beads [54] and FITC labeled bioparticles [5]; the present study used the latter [20] and found a suppressive effect of IRF4 on aged microglial phagocytosis, with IRF5 being irresponsible. Based on our knowledge, this is the first study that examined the involvement of IRF5 and IRF4 in microglial phagocytosis, and the result suggested that the anti-inflammatory IRF4 can hold phagocytosis in check to keep a balance of microglial phagocytic activity. Previous studies showed compared to young adult microglia, the aged microglia have an impaired phagocytic function at both baseline and after stimulus-induced activation [55], and the deficits in clearance by microglial phagocytosis have been implicated in the pathogenesis of age-related diseases such as Alzheimer’s [56, 57]. However, our data showed aged microglia have lower level of IRF4 than young adult microglia, which is not in line with the deficits in aged microglial phagocytosis found in these previous studies, suggesting other signals exist to also regulate the phagocytic activity. Several endogenous mechanisms have been found to control phagocytosis including signal regulatory protein-α (SIRPα), a receptor that is expressed primarily on myeloid cells and binds to its ligand CD47 and functions as a regulator of phagocytosis [58, 59]. Further studies are warranted to determine roles of IRFs in microglial phagocytosis.

Our data showed IRF4 or IRF5 had muted function either in the expression of some aged microglial cytokines (Supplementary Figure 2) or in phagocytosis (Supplementary Figure 3); however, they both impacted on stroke outcomes in a similar pattern as that in young mice [11]. The detrimental IRF5 and beneficial IRF4 data are consistent with their pro- or anti-inflammatory profile respectively; together with our previous studies on these IRFs with neonatal [32, 33, 44] and young adult [11, 12] stroke model, we conclude that IRF5-IRF4 regulatory axis regulate the post-stroke inflammation throughout the life span, and have significant effect on stroke outcomes. IRF5-IRF4 axis is a promising target for developing new, effective therapeutic strategies for stroke.

## MATERIALS AND METHODS

### Experimental animals

To generate mice lacking the IRF4 or IRF5 gene in microglia, we crossed LysMcre mice (strain # 018956; Jackson Laboratories, Bar Harbor, ME, USA) with IRF4 or IRF5 fl/fl mice. The validation of the IRF4 or IRF5 CKO has been performed in our previous studies [11]. Sham, IRF5 or IRF4 flox or CKO mice, were group-housed under pathogen-free conditions with a 12- to 12-h day-night cycle and had access to food and water *ad libitum*. Aged (18-20 months) and young (8-12 weeks) male mice were randomly chosen and used after they were examined free of aberrations or other abnormalities.

### Middle cerebral artery occlusion (MCAO) model

Stroke was induced by a 60-min reversible middle cerebral artery occlusion (MCAO) under isoflurane anesthesia as previously describe [60]. To occlude the MCA, 6.0-mm suture monofilaments (Doccol, Redlands, CA, USA), with silicone-coated tips were utilized in the diameter range 0.21 mm to 0.23 mm depending on the weights of mice. A midline ventral neck incision was made, and unilateral MCAO was performed by inserting the monofilament into the right internal carotid artery 6 mm from the internal carotid/pterygopalatine artery bifurcation via an external carotid artery stump. Reperfusion was performed by withdrawing the suture 60 min after the occlusion. To assess physiological parameters in mice, cerebral blood flow (CBF) was monitored by Laser Doppler Flowmetry (LDF, Moor Instruments Ltd, UK). Rectal temperature was maintained at 36.5 ± 0.5 ° C during surgery with an automated TC-1000 temperature-control feedback system (CWE, Inc., Ardmore, PA, USA). All mice were monitored on a daily base, and sacrificed at 3 d of reperfusion. Sham-operated animals underwent the same procedure including exposure to isoflurane, midline ventral neck incision, but the suture was not advanced into the MCA. Laser Doppler flow (Moor Instruments Ltd, UK) was applied to measure CBF through the skull at the right temporal fossa [61]. Only the mice whose CBF showed a drop of over 85% of baseline just after MCAO were included in the following experiments [62]. The mortality of aged IRF5/IRF4 CKO mice after the 60-min MCAO was 30%.

### Flow cytometry

Tissue processing for flow cytometry was performed as previously described [20, 54] with modifications. Briefly, phosphate-buffered saline perfused ipsilateral hemisphere of mouse brain, was placed in 5 mL of complete Roswell Park Memorial Institute (RPMI) 1640_302001 medium (American Type Culture Collection, Frederic, MD, USA) and supplemented with 10% FBS, 1% P/S, 150 μL collagenase/dispase (1 mg/mL), and 300 μL DNase (10 mg/mL). The brain was diced using a blade; the suspension was incubated for 45 min at 37° C, and with mild agitation. The cell suspension was further triturated and filtered through a 70-μm filter, and cells were washed and freed from myelin and brain tissue by 70-30% Percoll gradients separation. The cells were collected at the interface of the 70-30% Percoll gradients and washed with 1xPBS. For cell membrane inflammatory mediator staining, cells were immediately stained with live/dead cell discrimination Stain Kit_L34966 (1:1000 dilution, Invitrogen, USA) for 10 min after 1xPBS washing step; then blocked with mouse Fc Block_553142 (1:50 dilution; BD Bioscience, Franklin Lakes, NJ, USA) for 10 min after washing the cells in FACS buffer. Cells were then incubated with antibody-conjugated fluorophores including CD45-eF450_48045182, CD11b-AF488_47011282, Ly6C-APC-eF780_47593282 (Invitrogen, USA); Ly6G-PE_127608, CD 68-APC_137008, and CD206-PE-cy7_141720 (BioLegend, San Diego, CA, USA), at 1:50 dilution for 10 mins. After this step cells were washed in FACS buffer, then fixed in 2% PFA for 10 mins, then washed and resuspend in FACS ready for flow cytometry assessment.

For intracellular cytokine staining, an *ex vivo* brefeldin A protocol was followed [12, 63], and an intracellular antibody mixture at 1:50 dilution containing TNFα-PE-Cy7 (eBioscience, San Diego, CA, USA) and IL-1β-PE (eBioscience), IL-4-APC, and IL-10-PerCP-Cy5.5 (BioLegend), was used for staining. Fluorescence minus ones (FMOs) and beads compensations were used for all staining experiments for both cell membrane and intracellular inflammatory mediators. Data were acquired on Cytoflex-S (Beckman Coulter, Brea, CA, USA) or BD FACSMelody cytometers and analyzed using FlowJo (Treestar Inc., Ashland, OR, USA).

### Phagocytosis assay

For phagocytosis assays, FITC conjugated *E. coli*-derived (K-12 strain) bioparticles [20] were used. Briefly, brain cell suspension was processed as for flow cytometry, using 70-30% Percoll gradients. The cells were collected at the interface of the 70-30% Percoll gradients and washed with 1xPBS, then stained with live/dead cell discrimination Stain Kit_L34966 (1:1000 dilution, Invitrogen, USA) for 10 min after 1xPBS washing step; then blocked with mouse Fc Block_553142 (1:50 dilution; BD Bioscience, Franklin Lakes, NJ, USA) for 10 min and incubated with antibody-conjugated fluorophores CD45-eF450_48045182 and CD11b-eF780_47011280 (Invitrogen, USA), at 1:50 dilution and for 10 mins. After this step cells were washed and resuspended in FACS buffer for microglia sorting. To sort microglia-only cells we used BD FACSMelody™ cell sorter, and collected a maximum of <100,000 microglial cells per hemisphere. The collected microglial cells were concentrated at 1500 RMP at room temperature, and then resuspended in warm RPMI1640_302001 supplemented with 10% FBS, 1% P/S, and 0.01 mg/mL FITC conjugated *E. coli*-derived (K-12 strain) bioparticles (1 mL per assay), in cell culture tubes. The cells were then incubated in Forma Steri-Cycle CO_2_ incubator at 37° C, 95% humidity and 5% CO_2_, for 1 h. Cells were washed with FACS buffer, fixed with 2% PFA for 10 mins (if necessary), then washed and resuspend in FACS buffer for flow cytometry assessment.

### ELISA for plasma inflammatory mediator assay

Blood samples were obtained by cardiac puncture with heparinized needles (containing 65μL heparin) and centrifuged at 15000 RPM for 20 min and at 4° C. After centrifugation, the plasma was collected and stored at -80° C or used immediately with multiplex Enzyme-Linked Immunosorbent Assay (ELISA). We used the MultiPlex quantitative Bio-Plex Pro Mouse Cytokine 23-plex assay, according to the manufacturer’s instruction (# M60009RDPD, Bio-Rad Laboratories, Hercules, CA, USA). All assays were performed in duplicates, and the mean of each sample normalized (with a fit of the standards R^2^ = 0.98), using dilution factor based on the volume of heparin used (65 μL) and the final volume of blood collected.

### Corner test

Corner test was performed to measure the sensorimotor deficit as previously described [64, 65]. Briefly, the mouse was placed between two cardboard pieces (each 30 cm×20 cm×1 cm), and the boards were gradually moved closer to the mouse from both sides to encourage the mouse to enter into a corner of 30° with a small opening along the joint between the two boards. When the mouse entered the deepest part of the corner, both sides of the vibrissae were stimulated by the two boards. Then, the mouse reared forward and upward and turned back to face the open end. Twenty trials were performed for each mouse, and the percentage of right turns was calculated. Only turns involving full rearing along either board were recorded.

### Neurologic deficit scores (NDS)

Following MCAO, NDS were measured as: (0) no deficit; (1) forelimb weakness and torso turning to the ipsilateral side when held by tail; (2) circling to the affected side; (3) unable to bear weight on the affected side; and (4) no spontaneous locomotor activity or barrel rolling as previously reported [66]. The MCAO-induced infarct in the brain was analyzed with cresyl violet (CV) staining as described and measured in [57, 61].

### Statistical analysis

Data from individual experiments were presented as mean ± SD, and assessed by Student’s t test or 2-way ANOVA with Tukey post hoc test for multiple comparisons (GraphPad Prism Software 9.3.1 (471), San Diego, CA, USA). *P* < 0.05 was considered statistically significant. The ordinal data of NDS was analyzed with Mann-Whitney U test. Investigators were blinded to mouse strains for stroke surgery, behavioral testing, infarct, and inflammation analysis.

### Institutional review board statement

All studies were conducted in accordance with the United States Public Health Service’s Policy on Human Care and Use of Laboratory Animals, and all procedures were performed in accordance with NIH guidelines for the care and use of laboratory animals and approved by the Institutional Animal Care and Use Committee of The University of Texas Health Science Center at Houston and the McGovern Medical School.

### Data availability statement

The datasets used and/or analyzed in the present study are available from the corresponding author on reasonable request.

## Supplementary Material

Supplementary Figures
