# Supplementary material for: Regulation of microglial activation in stroke in aged mice: a translational study
Source: Aging (Albany NY). 2022 Aug 12;14(15):6047–65. doi: 10.18632/aging.204216 (PMC9417226; doi:10.18632/aging.204216)
Supplement: Supplementary Figures [file aging-14-204216-s001.pdf]

SUPPLEMENTARY FIGURES

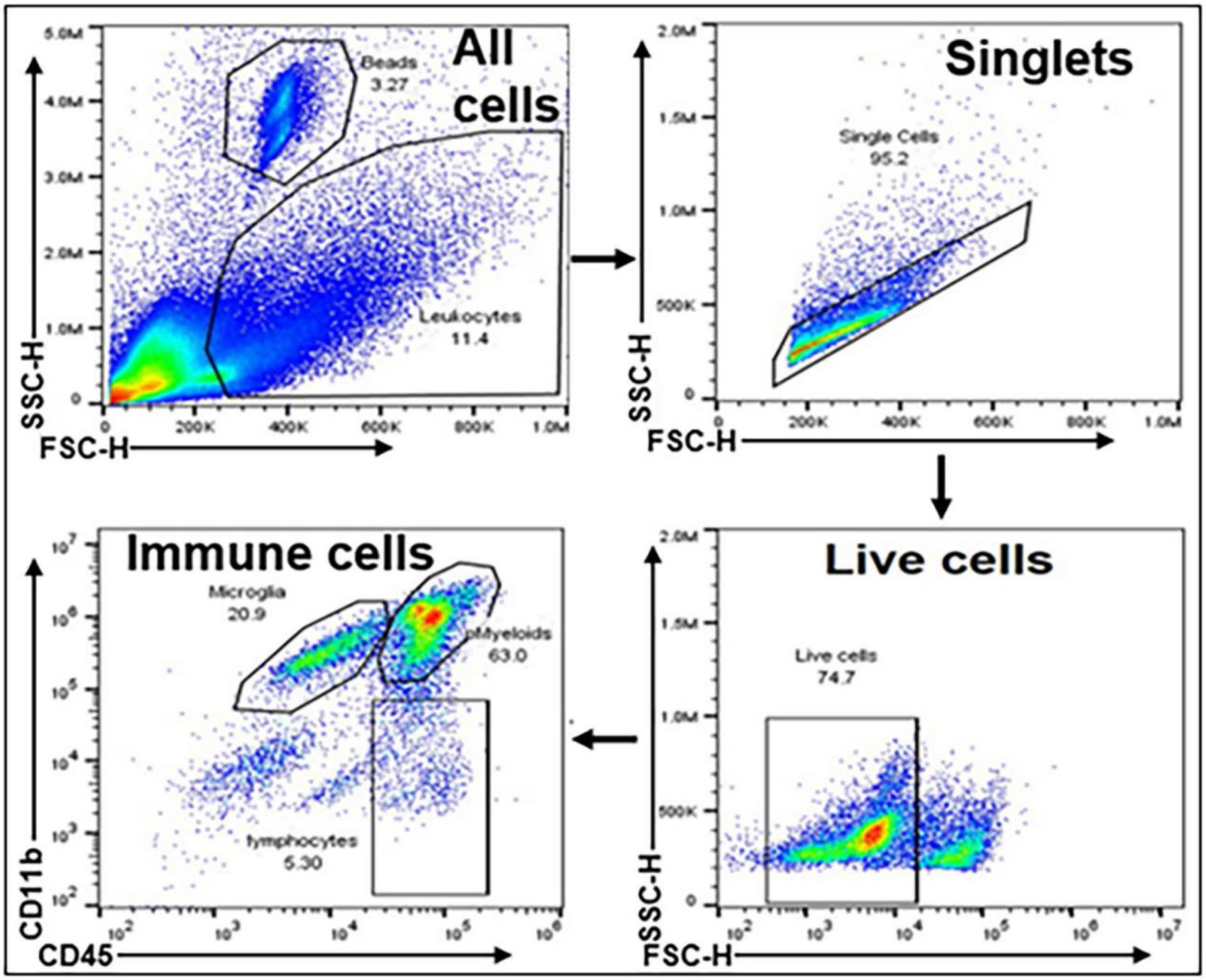

Supplementary Figure 1. Gating strategy to sequentially separate single cells, live cells, and leukocytes, including microglia.

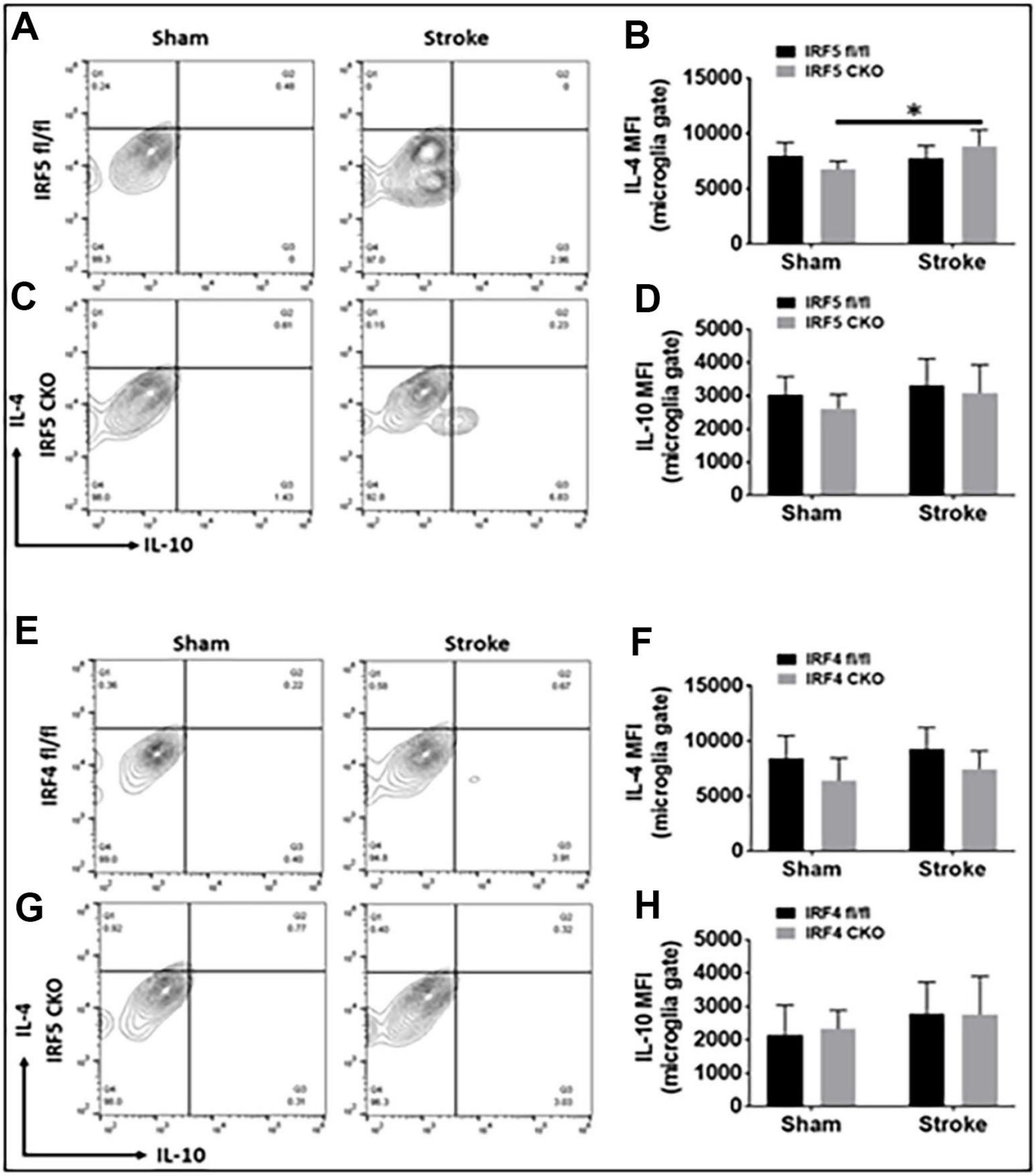

Supplementary Figure 2. Intracellular anti-inflammatory cytokine levels in IRF5 or IRF4 CKO vs. flox microglia by flow cytometry performed on stroke and sham brains. Quantification data are presented as mean MFI. (**A**–**D**) Data of IRF5 CKO and (**E**–**H**) data of IRF4 CKO microglia, respectively. (**A**, **C**) are representative intracellular staining plots for IL-4/IL-10 in both IRF5 CKO and flox microglia; (**E**, **G**) are plots for IL-4/IL-10 in both IRF4 CKO and flox microglia. MFI of these cytokines were quantified in (**B**, **D**, **F**, **H**). $n = 4$ to 5 per sham and 6 to 7 per stroke group; $^*P < 0.0500$.

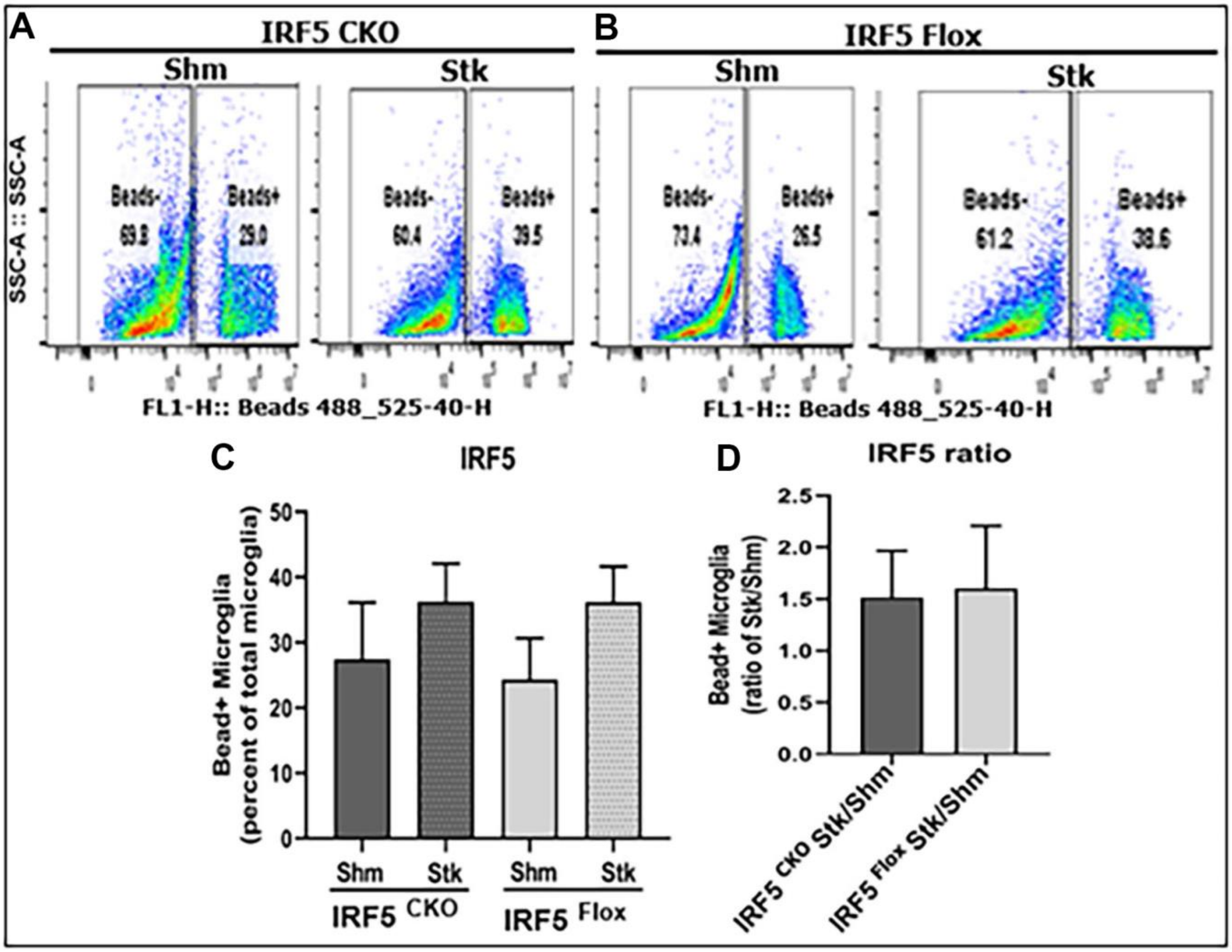

Supplementary Figure 3. Microglial phagocytosis by flow cytometry performed on IRF5 CKO stroke and sham mice. Quantification data are presented as mean percentage of bead+ microglia. (**A**, **B**) Fluorescence intensity plots for IRF5 KO and flox microglia exposed to FITC fluorescent bioparticles. (**C**) Percentage fluorescence of phagocytosis in IRF5 CKO vs. flox sham and stroke microglia; and (**D**) comparative quantified data for the ratio of IRF5 CKO stk/shm vs. IRF5 flox stk/shm in (**C**). *n* = 4 to 5 per sham and 6 to 7 per stroke group.
